# Erythrodermic Psoriasis Causing Uric Acid Crystal Nephropathy

**DOI:** 10.1155/2019/8165808

**Published:** 2019-03-28

**Authors:** John Ellis, Jeffrey Lew, Sumir Brahmbhatt, Sarah Gordon, Troy Denunzio

**Affiliations:** ^1^Department of Medicine, Tripler Army Medical Center, Honolulu, HI, USA; ^2^Nephrology Service, Tripler Army Medical Center, Honolulu, HI, USA

## Abstract

**Background:**

Erythrodermic psoriasis is a rare and severe variant of psoriasis. It is characterized by widespread skin erythema, scaling, pustules, or exfoliation of more than 75% of the body's surface area. This condition has life-threatening complications to include hemodynamic, metabolic, immunologic, and thermoregulatory disturbances. One metabolic complication, hyperuricemia, occurs from rapid keratinocyte differentiation and infiltration of inflammatory cells into psoriatic lesions. Although renal injury caused by shunting of blood to the skin has been reported, there are no reports of erythrodermic psoriasis causing crystal-induced nephropathy. We present a case of erythrodermic psoriasis and hyperuricemia complicated by uric acid crystal nephropathy.

**Case Presentation:**

A 57-year-old male with long-standing psoriatic arthritis presented with diffuse scaling of his skin. He was being treated with adalimumab, leflunomide, and topical clobetasol, but had recently stopped taking his medications. Physical exam revealed yellow scaling covering his entire body with underlying erythema and tenderness without mucosal involvement. Labs were notable for a creatinine of 3.3 mg/dL, with no prior history of renal disease, and uric acid of 12.7 mg/dL. He was admitted to the intensive care unit given >80% of body surface area involvement and acute renal failure. Despite aggressive fluid resuscitation, renal function did not improve, and creatinine peaked at 4.61 mg/dL. Urine microscopy showed diffuse polymorphic uric acid crystals, consistent with uric acid crystal-induced nephropathy. He was started on rasburicase, urinary alkalinization, and fluids. His renal function improved dramatically; urine output, uric acid, and electrolytes normalized. He was discharged on topical clobetasol and leflunomide and started on secukinumab with little to no skin involvement.

**Conclusion:**

This case presents the rare complication of crystal-induced nephropathy in a patient with erythrodermic psoriasis. Uric acid crystal nephropathy is well described in diseases with rapid cell turnover such as tumor lysis syndrome. It is thought that rapid keratinocyte differentiation and inflammatory infiltration of psoriatic lesions produced life-threatening electrolyte abnormalities similar to tumor lysis syndrome. Early recognition of this rare complication is critical, and aggressive fluid resuscitation, urine alkalinization, and uric acid lowering agents should be administered immediately.

## 1. Background

Erythrodermic psoriasis (EP) is a rare and severe variant of psoriasis that occurs in 1-2% of affected patients. It is characterized by widespread skin erythema, scaling, pustules, or exfoliation of more than 75% of the body's surface area [1]. There are multiple documented and emerging causes of EP. The main risk factors are history of psoriasis and medications. In a retrospective review of 50 patients with EP, the mean time of diagnosis of psoriasis to EP flare was 14 years and rarely was EP, the presenting symptom for psoriasis diagnosis [2]. In addition, there is a relationship between EP and starting certain medications including drugs used to treat psoriasis. It has been well documented that starting oral or topical corticosteroids for psoriasis can cause an EP flare as can abruptly discontinuing corticosteroids for treatment of psoriasis [2]. Similarly, the use of biologics has been reported as causing EP in patients being treated for psoriasis and Crohn's disease [3, 4], and there are several case reports of abrupt cessation of biologic agents precipitating EP [5, 6].

EP has life-threatening complications to include hemodynamic, metabolic, immunologic, infectious, and thermoregulatory disturbances. The erythroderma causes exfoliative dermatitis of the skin, shedding the epidermis and increasing the risk of *Staphylococcus aureus* septicemia [7]. The generalized inflammation of the skin causes vasodilation and subsequent thermoregulatory disturbances. The shunting of the blood combined with the exfoliative losses causes increased transpiration and fluid losses proportional to the surface area involved. As a result of the fluid losses and shunting, there are multiple electrolyte abnormalities and organ hypoperfusion effects that can occur. One metabolic complication that has been reported in EP is hyperuricemia, which has both acute and chronic components. Chronically elevated uric acid occurs from rapid abnormal keratinocyte differentiation and infiltration of inflammatory cells into psoriatic lesions. These lead to increased cell turnover and nucleic acid breakdown [8]. Additionally, psoriasis itself can cause immune-related IgA deposits from T-cell activation and tumor necrosis factor-*α* production causing psoriatic nephropathy [9]. EP can be associated with acute kidney injury from medications or hypoperfusion as described above which can reduce the clearance of uric acid.

With multiple acute on chronic causes of hyperuricemia, there are no reports of EP causing crystal-induced nephropathy. The most common causes of uric acid nephrolithiasis include primary gout, gastrointestinal disorders, neoplastic disorders, diet, and sometimes inherited metabolic abnormalities [10, 11]. We present a unique case of erythrodermic psoriasis and hyperuricemia complicated by uric acid crystal nephropathy and acute kidney injury.

## 2. Case Presentation

**Figure 1:**
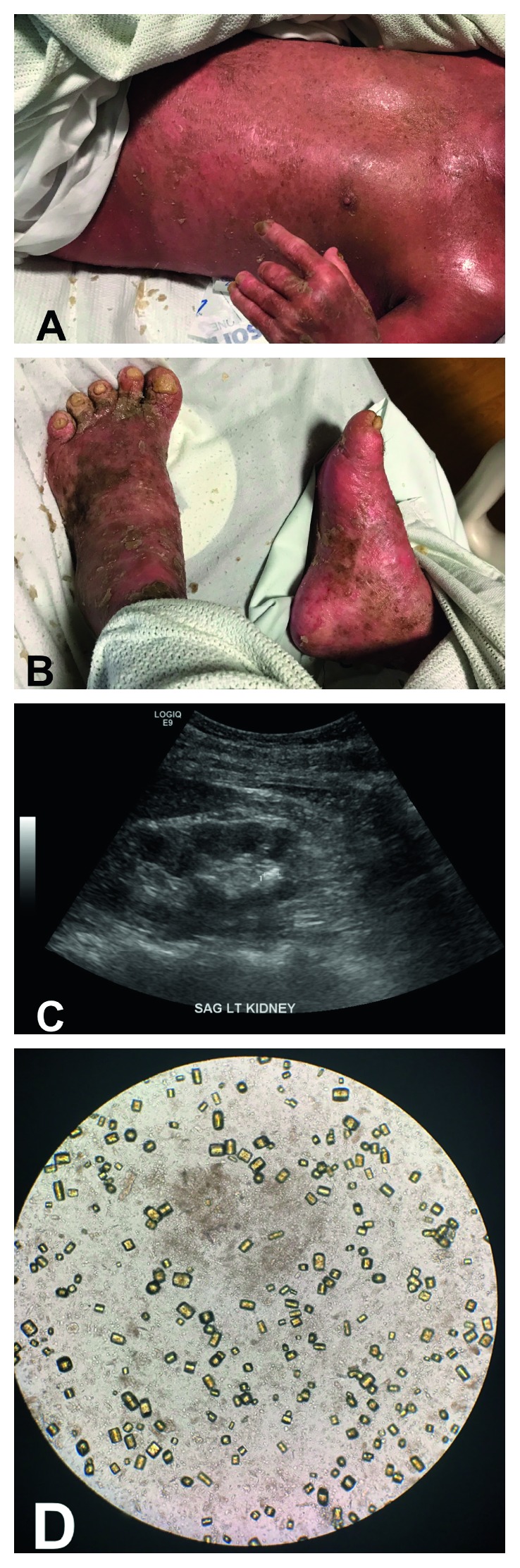
(a) The patient with exfoliative dermatitis with severe erythroderma on the upper torso. (b) Skin lesions also affecting lower extremities with nail involvement. (c) Renal ultrasound showing nephrolithiasis in the renal calyx. (d) Urine sediment displaying numerous rounded parallelogram‐shaped crystals, consistent with uric acid crystals.

A 57-year-old male from Thailand with long-standing psoriatic arthritis presented with diffuse scaling of his skin. He was being treated for psoriatic arthritis with adalimumab, leflunomide, and topical clobetasol, but had stopped taking all medications two weeks prior to presentation. At baseline, the patient was a gardener and able to complete all his activities of daily living. However, during the disease course, he noticed diffuse skin scaling and had diarrhea, leading to fatigue. His only joint complaint was chronic left ankle pain, and he denied oral ulcers. Physical exam revealed diffuse yellow scaling covering his entire body with underlying erythema and tenderness without mucosal involvement (Figures 1(a) and 1(b)). Empiric antibiotics were started until blood cultures ruled out infection. Labs were notable for a creatinine of 3.3 mg/dL, with no prior history of renal disease, calcium 7.8 mg/dL, phosphate 5.9 mg/dL, bicarbonate 13.0 mmol/L, urine pH 5.0, and uric acid of 12.7 mg/dL (Table 1). The patient was admitted to the intensive care unit given >80% of body surface area involvement and acute renal failure. He received 4 L of normal saline for initial resuscitation. On day 2, the patient was determined to be euvolemic based on clinical exam with moist mucous membranes. Despite aggressive fluid administration, renal function did not improve, and creatinine continued to rise and peaked at 4.61 mg/dL, with phosphate of 7.0 mg/dL and 48 hours of anuria. Renal ultrasound showed 5 mm nonobstructing renal calculi in the left kidney and small echogenic kidneys consistent with chronic kidney disease (Figure 1(c)). Subsequent urine microscopy showed diffuse polymorphic uric acid crystals, consistent with uric acid crystal-induced nephropathy (Figure 1(d)). He was managed with rasburicase, urinary alkalinization, and fluids. The underlying erythrodermic psoriasis was treated with restarting leflunomide, topical isotretinoin, and clobetasol. His renal function improved dramatically; urine output, uric acid, and electrolytes normalized. He was discharged on topical clobetasol and leflunomide and new addition of secukinumab with little to no skin involvement.

## 3. Conclusions

Uric acid crystal-induced nephropathy is a rare complication of severe erythrodermic psoriasis. Although hyperuricemia has been reported in patients with psoriasis, uric acid-induced crystal nephropathy has not been reported in the setting of EP. In a study comparing uric acid levels in different types of psoriasis, EP had the highest serum uric acid levels and serum creatinine compared to psoriasis vulgaris and generalized pustular psoriasis [12]. However, the exact etiology for this hyperuricemia is unknown. In our case, it is thought to be due to a multifactorial process that caused uric acid elevation from the psoriasis and renal failure. Psoriasis has increased infiltration of inflammatory cells in addition to rapid cellular turnover producing a tumor lysis syndrome-like picture [13]. The patient's renal failure was likely due to prerenal azotemia from decreased oral intake, diarrhea and fluid losses from his skin resulting decreasing the excretion of uric acid and production of urine resulting in a microenvironment ideal for uric acid nephrolithiasis.

Treatment of EP with uric acid-induced nephrolithiasis includes both treating the underlying EP and treatment of the uric acid nephrolithiasis. For EP, treatment is difficult because there are no large double-blind placebo-controlled randomized trials due to low incidence. Through nonrandomized controlled trials and case series, first-line treatment for EP includes cyclosporine, infliximab, topical retinoids and methotrexate, and supportive management [1]. However, each of these agents should be used with extreme caution. Recognizing uric acid nephrolithiasis and acute kidney injury influences selection of EP treatment because cyclosporine and methotrexate should be avoided in patients with CrCl <30 ml/min. In addition, cyclosporine has been documented numerous times to increase serum uric acid levels which can exacerbate uric acid nephrolithiasis [14]. Selection of infliximab should also be used with caution because *Staphylococcus aureus* infection can both precipitate and exacerbate EP flares as immunosuppression would put patients at greater risk for septicemia [7, 15].

Uric acid-induced nephropathy treatment was individualized for this patient's underlying hyperuricemia to include aggressive fluid resuscitation, urine alkalinization, and uric acid lowering agents. Although uric acid nephrolithiasis can be treated with macromolecular inhibitors of crystallization such as rasburicase, and pegloticase, it has been mostly studied in patients with tumor lysis syndrome specifically in childhood leukemias and lymphoma [16]. These medications have not been studied in the context of hyperuricosemia from EP. However, due to the rapidity and severity of symptoms and electrolyte abnormalities similar to tumor lysis syndrome, our patient was treated with rasburicase with clinical and laboratory improvement.

It is critical for providers to suspect hyperuricemia and uric acid-induced nephrolithiasis when a patient presents with EP. Treatment should be individualized to the patient, and if renal function does not improve after fluid resuscitation, there should be a low threshold for continued workup to include renal ultrasound, urine microscopy, and serial electrolyte panels, as well as initiation of a uric acid lowering agent such as rasburicase since the patient is at high risk for progression to life-threatening complications and progression to kidney failure.

## Figures and Tables

**Table 1:** Presenting labs.

Initial studies	Presentation			Hospital day 3		
Basic metabolic panel	Na	138	(136–145) mmol/L	Na	135	(136–145) mmol/L
	K	5.1	(3.7–5.3) mg/dL	K	4.6	(3.7–5.3) mg/dL
	Cl	110	(98–107) mmol/L	Cl	100	(98–107) mmol/L
	HCO_3_	13	(22–29) mmol/L	HCO_3_	18	(22–29) mmol/L
	BUN	37	(7–23) mg/dL	BUN	38	(7–23) mg/dL
	Cr	3.30 H	(136–145) mg/dL	Cr	4.61 H	(136–145) mg/dL
	Glucose	69	(70–105) mg/dL	Glucose	231	(70–105) mg/dL
	Ca	7.8 (L)	(8.4–10.2) mg/dL	Ca	7.6 (L)	(8.4–10.2) mg/dL
	Mg	1.5	(1.6–2.6) mg/dL	Mg	2.0	(1.6–2.6) mg/dL
	P	5.9 (H)	(2.3–4.7) mg/dL	P	5.9 (H)	(2.3–4.7) mg/dL
	Alb	2.0 (L)	(3.5–5.0) g/dL	Alb	2.1 (L)	(3.5–5.0) g/dL

Uric acid	12.5		(2.6–7.2) mg/dL		13.1	(2.6–7.2) mg/dL

Urinalysis	Color		Yellow	Color		Yellow
	Appearance		Hazy	Appearance		Hazy
	SG		1.018 (1.003–1.030)	SG		1.020 (1.003–1.030)
	pH		5.0 (4.5–8.0)	pH		5.0 (4.5–8.0)
	Protein		+1 (H) (negative)	Protein		+1 (H) (negative)
	Ketones		Trace	Ketones		Trace
	Bilirubin		Negative (negative)	Bilirubin		Negative (negative)
	Urobiligen		Normal (0.2–1.0) mg/dL	Urobiligen		Normal (0.2–1.0) mg/dL
	UA blood		+2 (negative)	UA blood		+2 (negative)
	Leuk		Est negative	Leuk est		+2 (H)
	RBC UA		10 (H) (0–5)/HPF	RBC UA		10 (H) (0–5)/HPF
	WBC		6 (H) (0–5)/HPF	WBC		6 (H) (0–5)/HPF
	Bacteria		Rare	Bacteria		Rare
	Squamous cells		2	Squamous cells		2

Urinary eosinophils				Negative		

Renal ultrasound	Left kidney 9 cm and lower pole renal calculi 5 mm nonobstructing. Right kidney 8 cm renal cortices echogenic bilaterally consistent with medical renal disease.					

## Abbreviations

EP: Erythrodermic psoriasis
FENa: Fractional excretion of sodium.
